# Charactering Neural Spiking Activity Evoked by Acupuncture Through Coupling Generalized Linear Model

**DOI:** 10.3390/e26121088

**Published:** 2024-12-13

**Authors:** Qing Qin, Kaiyue Zhang, Yanqiu Che, Chunxiao Han, Yingmei Qin, Shanshan Li

**Affiliations:** Tianjin Key Laboratory of Information Sensing & Intelligent Control, Tianjin University of Technology and Education, Tianjin 300222, China; qing@tute.edu.cn (Q.Q.); kyzhang@tute.edu.cn (K.Z.); yqche@tju.edu.cn (Y.C.); cxhan@tju.edu.cn (C.H.); eeymqin@tju.edu.cn (Y.Q.)

**Keywords:** acupuncture, spike history, coupling, generalized linear model, maximum likelihood estimation

## Abstract

Acupuncturing the ST36 acupoint can evoke a responding activity in the spinal dorsal root ganglia and generate spikes. In order to identify the responding mechanism of different acupuncture manipulations, in this paper the spike history of neurons is taken as the starting point and the coupling generalized linear model is adopted to encode the neuronal spiking activity evoked by different acupuncture manipulations. Then, maximum likelihood estimation is used to fit the model parameters and estimate the coupling parameters of stimulus, the self-coupling parameters of the neuron’s own spike history and the cross-coupling parameters of other neurons’ spike history. We use simulation data to test the estimation algorithm’s effectiveness and analyze the main factors that evoke neuronal responding activity. Finally, we use the coupling generalized linear model to encode neuronal spiking activity evoked by two acupuncture manipulations, and estimate the coupling parameters of stimulus, the self-coupling parameters and the cross-coupling parameters. The results show that in acupuncture experiments, acupuncture stimulus is the inducing factor of neuronal spiking activity, and the cross-coupling of other neurons’ spike history is the main factor of neuronal spiking activity. Additionally, the higher the amplitude of the neuronal spiking waveform, the greater the cross-coupling parameter. This lays a theoretical foundation for the scientific application of acupuncture therapy.

## 1. Introduction

By stimulating the receptors in the acupoint area, acupuncture evokes responses in the nervous system and then affects the target organs [1,2]. Different acupuncture manipulations stimulate the acupoint areas, and the nervous system will evoke different responding activity, and finally achieve different regulatory effects on the human body [3,4]. However, the responding mechanism of the nervous system to different acupuncture manipulations is still unclear. Therefore, different acupuncture manipulation experiments are designed to detect the response of acupuncture nerve circuits and reveal the relationship between the stimulus, coupling relationship and neuronal spiking activity. It will be helpful to understand the responding mechanism of different acupuncture manipulations and provide a theoretical basis for acupuncture learning and clinical treatment.

Neuronal model analysis is an important method for studying the encoding of neural information. The neurons convert the time-varying input signals into precise spiking trains [5,6], and the dynamic physiological mechanism of this process has gained wide attention from researchers. In the early studies of neural spiking activity, the famous Hodgkin–Huxley (H-H) model laid a critical theoretical foundation for subsequent research on neuronal information encoding by describing the biophysical mechanisms underlying the generation and propagation of neuronal spikes [7]. Later, Hindmarsh and Rose proposed the Hindmarsh–Rose (H-R) model, which is a mathematical model used to simulate neuronal spikes. The H-R model not only reproduces diverse neuronal spikes, but also provides significant theoretical support for exploring complex mechanisms of neural information encoding [8,9]. In addition, some phenomenological models [10,11,12] entered the field of view, some of which were highlighted in the simulation experiments that fit the recorded data of electrophysiological experiments [12,13,14]. The phenomenological model simulated the process of generating spikes through simple mathematical equations according to the spiking characteristics of neurons. In 1907, Lapicque proposed the IF model that provided a simple mechanistic explanation for the basic behavior of neurons [15]. As a typical phenomenological model, the Leaky integrate-and-fire (LIF) model takes the integrate-and-fire (IF) model as its theoretical basis.

The changes in the membrane potential of neurons and the spiking data both have intrinsic statistical regularities. When choosing a neuronal encoding model, it is necessary to consider the flexibility and the fitting ability of the model, as well as the interpretability of the parameters, while the model also needs to be compatible with the knowledge of the physiology and anatomy of the nervous system. The generalized linear model (GLM) [16,17] has unique advantages in the research of the nervous system, which can explain the parameters and the predicted results of the model from both statistical and biophysical levels. Bernander et al. used GLM to construct a neural encoding model based on conductance and successfully predicted the synaptic input corresponding to the spike [18]. Pillow et al. used GLM to model retinal ganglion cells and predicted the spiking trains of the stimulus–response of single ganglion cells [19]. Calabrese et al. improved the GLM by adding the influence of the neuron’s own spike history on neuron spiking activity into the model, and added the spike-history filter to predict the stimulus–response trains of auditory neurons [20]. Wei et al. used GLM to fit the spiking data of spinal dorsal horn of mouse and reconstructed the mapping from acupuncture stimulus to spiking data [21]. However, the neuronal spiking activity is not only affected by the spike history of self, but also by the spike history of neighboring, coupled neurons [17]. In early studies, the coupling effects between neurons were not fully considered. As the research progressed, some scholars began to introduce dynamic dependencies between neurons in neuronal encoding models [22,23], and studies have found that the spike history of neighboring neurons also plays an important role in neuronal spiking activity [24,25,26]. At present, GLM and its improved models are widely used in the study of nervous systems and neuronal spikes, realizing the accurate fitting and prediction of experimental data.

Acupuncture is an external mechanical stimulus with a low stimulus frequency. Each stimulus can evoke a dense cluster of neuronal spikes, and these spikes become spike history, which will evoke more subsequent spikes. Therefore, spike history is an important factor that cannot be ignored in acupuncture spiking response. In this paper, the spike history of neurons is taken as the starting point for studying the encoding mechanism of acupuncture neurons according to the ideas of Pillow et al. We model the dynamic dependence between acupuncture neurons as a linear coupling of the spike history, which is added to the GLM to obtain the coupled encoding model of acupuncture neurons. Meanwhile, the classic maximum likelihood estimation (MLE) is used to estimate the coupling parameters of the model. According to the estimated results, the experimental phenomenon is explained scientifically and the responding mechanisms of different acupuncture manipulations are revealed.

## 2. Materials and Methods

### 2.1. Generalized Linear Model

In the simplest linear–non-linear–Poisson (LNP) model, the neuronal spiking activity can be simulated by a non-linear combination of the response outputs of a series of linear filters. The spike-history terms are not included in this model, assuming that the spiking trains are generated by an inhomogeneous Poisson process, the spiking rate generated in this model is as follows:

(1) $$\lambda (t)=f(\vec{k}\cdot \vec{x}(t))$$

where the stimulus vector $\vec{x}(t)$ at time t passes through the linear stimulus filter $\vec{k}$, and then through the non-linear filter f to produce the spiking rate λ(t).

A key goal in constructing the encoding models is to relate the model parameters to biophysics and physiology. We add the neuron’s own spike history to the LNP model to obtain a soft-threshold integrate-and-fire model [27]. At this time, the neuronal spiking rate is the following:

(2) $$\begin{array}{l} \lambda (t)=f(\vec{k}\cdot \vec{x}(t)+\vec{h}\cdot \vec{y_{hist}}(t))\\ \;\;\;\;\;\;\;\;=\exp(\vec{k}\cdot \vec{x}(t)+\vec{h}\cdot \vec{y_{hist}}(t)). \end{array}$$

where the spike history ${\vec{y}}_{hist}(t)$ is added to the linear filtering and its filter is $\vec{h}$. The non-linear function f(⋅) is defined as an exponential transform [28].

In the neuronal spiking activity of acupuncture, the effect of other neurons’ spike history will also be evoked, which is not reflected in the above model. We adopt GLM that incorporates its own and neighboring neurons’ spike history [26,29], denoted as $\vec{y}(t)$. The spiking rate of the neuron i at time t is given by the following equation:

(3) $$\begin{array}{l} \lambda_{i}(t)=f(\vec{k_{i}}\cdot \vec{x}(t)+\sum_{j}{\vec{h}}_{ij}\cdot {\vec{y}}_{j}(t))\\ \;\;\;\;\;\;\;\;=\exp(\vec{k_{i}}\cdot \vec{x}(t)+\sum_{j}{\vec{h}}_{ij}\cdot {\vec{y}}_{j}(t)). \end{array}$$

where ${\vec{h}}_{ij}$ is a linear filter of the spike history; i=j denotes the self-coupling of the neuron i and i≠j denotes the cross-coupling of other neurons.

### 2.2. Classical Maximum Likelihood Estimation

In computational neuroscience, we need to determine the association between external variables (e.g., sensory stimulus or motor behavior) and neuronal spiking activity. The neuronal spiking process can be regarded as a Poisson random process, we need to describe this relationship statistically, by predicting the probability $p(Y|\vec{x})$ of the specific neuronal spiking train Y given the external stimulus signal $\vec{x}$. The stimulus vector $\vec{x}(t)$ includes stimuli present at time t. We cannot directly estimate the probability $p(Y|\vec{x})$ of the stimulus–response pair $\left(\vec{x}|Y\right)$; therefore, we assume the conditional probability $p(Y|\vec{x},\theta )$ and fit the model parameter θ to the observed data. Since the model proposed in this paper is the coupling GLM, the model parameters are estimated using the MLE, which is widely used in GLMs [30,31].

In the LNP model, the parameter is $\theta =\vec{k}$. Then, we added the effects of the spike-history trains of other neurons to the model. Thus, the GLM is obtained that not only analyzes the spike-history trains of individual neurons, but also encompasses the common encoding between neurons [32,33]. At this point, the model parameter is $\theta =\left\{\vec{k},\vec{h}\right\}$.

To estimate the model parameters, we define the likelihood function of this model as $p(Y|\theta ,\vec{x})$, and then use the MLE to obtain the model parameters. In this model, the spiking trains follow the Poisson distribution, and probability of evoking $n_{t}$ spikes in a time window is the following:

(4) $$p[N(t+\Delta t)-N(t)]=\frac{{\left[\lambda (t)\cdot \Delta t\right]}^{n_{t}}\cdot \exp[-(\lambda (t)\cdot \Delta t)]}{n_{t}!}.$$

The probability that entire spiking trains is evoked is as follows:

(5) $$p[Y|X,\theta ]=\Pi \frac{{\left[\lambda (t)\cdot \Delta t\right]}^{n_{t}}\cdot \exp[-(\lambda (t)\cdot \Delta t)]}{n_{t}!}.$$

The log-likelihood function is the following:

(6) $$\log p(Y|X,\theta )=c+\sum \left[n_{t}\log(\lambda (t)\cdot \Delta t)-\lambda (t)\cdot \Delta t\right].$$

where X is the matrix of observed stimuli; Δt is the width of the time window of the observed spiking trains; c is a constant that does not depend on the parameter θ and $n_{t}$ is the number of spiking events in the time window Δt.

## 3. Results

### 3.1. Simulation Data Analysis

In this section, simulation data will be used to test the accuracy of the estimation method. At the same time, the main factors affecting neuronal spiking activity will also be discussed.

#### 3.1.1. Testing the Accuracy of the Estimation Method

We verify the accuracy of MLE with a set of simulation data. First, we use the known model to generate simulated spiking trains, where the parameters of the model are the true values in Table 1. Then, MLE is used to fit the simulated spiking trains to the model with unknown parameters, estimating the parameters of the model. The estimated values are shown in Table 1. For neuron i, $k_{i}$ denotes the coupling parameters of stimulus; $h_{ii}$ represents the self-coupling parameter of its own spike history and $h_{ij}$ represent the cross-coupling parameters of other neurons’ spike history (i=1, 2, 3).

Comparing the true and estimated values in Table 1, we find that the relative error of the estimated coupling parameters is distributed in the range of 0.14% to 3.76%, and the result has a 95% confidence level. Through the analysis, MLE can effectively estimate the parameters of this model.

**Figure 1:**
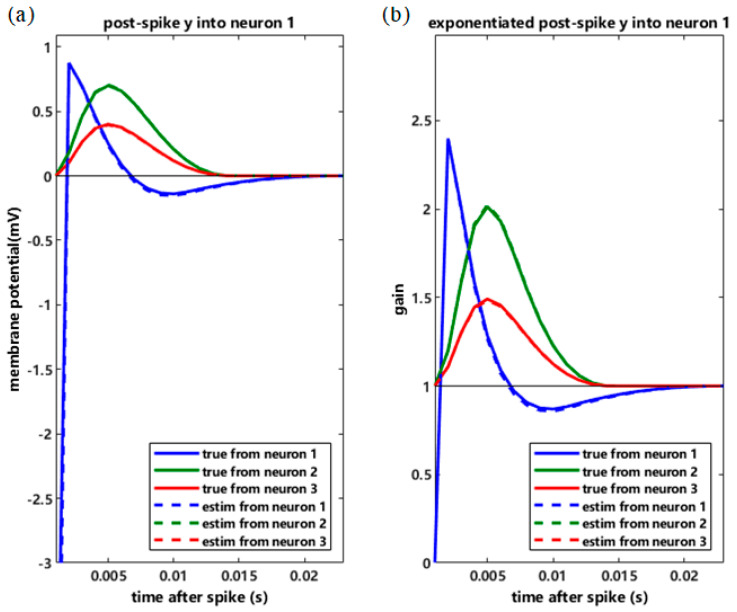
The effect of spike history of three neurons on the spiking activity of neuron 1.

In order to further verify the effectiveness of MLE, we take neuron 1 as an example. Figure 1a shows the effects of the spike history of three neurons on the spiking activity of neuron 1, and Figure 1b shows the effects of the spike history of three neurons after exponential transformation. The solid lines represent the effects of the spike history of neuron 1 itself and the other neurons generated by the known model. The estimated effects of the spike history of neuron 1 itself and other neurons are shown as dashed lines in Figure 1. Through Figure 1, we can conclude that the waveforms of the effects of neuronal spike history obtained by MLE can well capture the dynamic changes in the waveforms of the effects of actual neuronal spike history.

#### 3.1.2. The Main Factors Evoking Neuronal Spiking Activity

**Figure 2:**
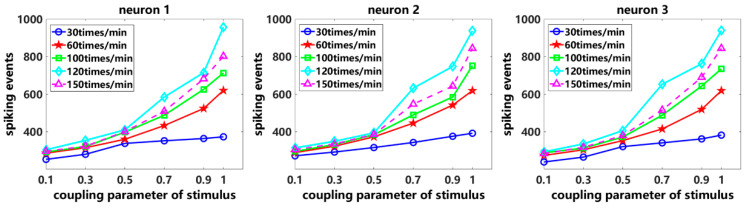
Numbers of neuronal spiking events by varying the coupling parameter of stimulus.

**Figure 3:**
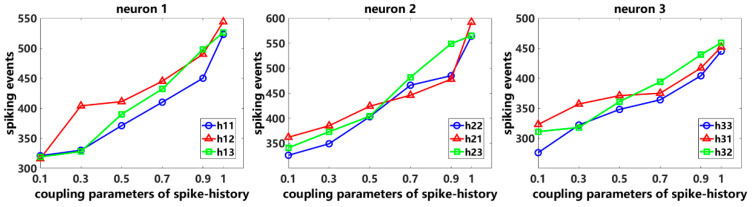
Numbers of neuronal spiking events by varying the coupling parameters of spike history.

Neuronal spiking activity is evoked by multiple factors, such as the input stimulus (external mechanical stimulus or internal current stimulus), the neurons’ own spike history, and the spike history of neighboring, coupled neurons [17]. In this section, we use simulation data to discuss the main factors that evoke neuronal spikes. We study the effects of external stimulus and spike history on neuronal spiking activity by changing the stimulus frequency, the coupling parameter of stimulus and the coupling parameters of spike history, and statistically analyze the spiking events of three neurons. The results are shown in Figure 2 and Figure 3.

First, with the coupling parameters of spike history being constant, we vary the stimulus frequency and the coupling parameter of stimulus. The stimulus frequencies used are 30 times/min, 60 times/min, 100 times/min, 120 times/min and 150 times/min, and the coupling parameter of stimulus gradually increases from 0.1 to 1. Taking neuron 1 in Figure 2 as an example, we find that with the increase in the coupling parameter of stimulus, the number of neuronal spiking events gradually increases. In addition, the stimulus frequency gradually increases from 30 times/min to 120 times/min, and the amplitude of the slope of the curve increases significantly, which means more neuronal spikes are evoked. However, when the stimulus frequency is increased to 150 times/min (magenta dashed line in Figure 2), the number of neuronal spikes does not exceed the number of neuronal spikes evoked by the stimulus frequency of 120 times/min. The same experimental phenomenon is observed for neuron 2 and neuron 3. This occurs because when the stimulus frequency is too high, the release of ion channels and neurotransmitters can reach the saturated state, preventing the evocation of additional neuronal spikes. This phenomenon is a self-protection mechanism of neurons, ensuring that neurons do not become hyperactive and become damaged under high-frequency stimulation. The coupling GLM effectively characterizes this neuronal property. Through the above analysis, we conclude that the characteristics of the stimulus itself, such as the stimulus frequency, and the coupling characteristics of stimulus, such as the coupling parameter, are the main factors that affect the neuronal spiking activity. When the stimulus frequency increases to a certain extent, due to the neuronal frequency adaptation and saturation mechanism, no additional neuronal spikes are evoked.

Then, under the condition that the stimulus frequency is 100 times/min and the coupling parameter of stimulus remains unchanged, the self-coupling parameters and the two cross-coupling parameters of spike history are gradually increased from 0.1 to 1, respectively. The results are shown in Figure 3. For neuron 1, when the self-coupling parameter $h_{11}$ or the cross-coupling parameters $h_{12}$ or $h_{13}$ of the spike history are gradually increased, the number of neuronal spikes also gradually increases with the rise of coupling parameters. This phenomenon is also observed in the spiking activity of neurons 2 and 3. In general, enhancing the self-coupling parameters or cross-coupling parameters of neuronal spike history can also evoke more neuronal spiking activity. In other words, the neuron’s own spike history as well as the other neurons’ spike history are also the main factors affecting the neuronal spiking activity.

### 3.2. Acupuncture Data Analysis

In the acupuncture experiment, we selected the widely used ST36 acupoint as the object of acupuncture stimulus, which is located on the lateral side of the tibia below the knee. In the experiment of peripheral nerve (spinal dorsal root experiment), the experimental subjects are healthy adult male Sprague-Dawley (SD) rats weighing about 200 g. First, the rats are anesthetized, the nerve bundles are separated, and the nerve fibers corresponding to the ST36 acupoint at the spinal dorsal root are picked out and implanted with bipolar platinum electrodes. Then, when the nerve fibers are resting, the acupuncture stimulus is applied to the ST36 acupoint. We adopt the following two common manipulations in the clinical treatment: twirling manipulation, and lifting–thrusting manipulation. The stimulus frequency is 100 times/min and the duration of stimulus for each manipulation is one minute.

First, the neuronal spiking waveforms are extracted and classified for the cluster spiking activity recorded by the electrodes, and all collected spikes are classified into corresponding neurons [34]. Most spiking classification algorithms are based on spiking waveforms, but the superposition of spiking waveforms [35,36,37] and the background noise [38,39,40] can cause deformation of spiking waveforms, and the classification effect will become worse. In order to effectively identify the variant spiking waveforms, reduce the leakage rate of the spike, and improve the accuracy of classification results. We take the results of the wavelet clustering algorithm [41,42] as the initial value and use the Bayesian classification algorithm to optimize the classification results [43,44].

**Figure 4:**
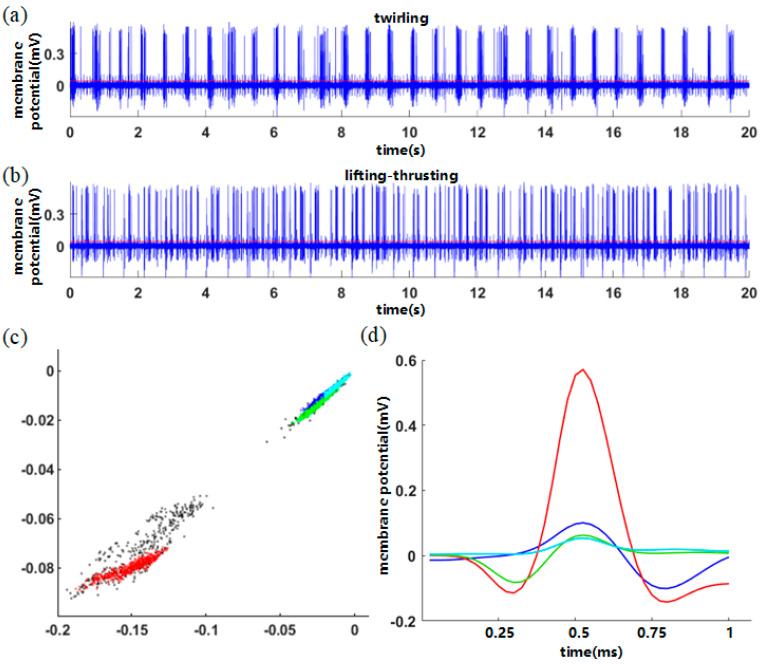
Spiking classification. Raw data evoked by the two acupuncture manipulations: (**a**) “twirling manipulation”; (**b**) “lifting−thrusting manipulation”; (**c**) the two−dimensional map of wavelet coefficients for all spiking waveforms; (**d**) four average spiking waveforms in the classification results: neuron 1 (red), neuron 2 (blue), neuron 3 (green) and neuron 4 (cyan).

The classification case is shown in Figure 4. Figure 4a,b show the raw data evoked by two acupuncture manipulations, the twirling manipulation and lifting–thrusting manipulation. Figure 4c shows the wavelet characteristic parameters of all spiking waveforms under the two manipulations, which is a two-dimensional scatter plot. The wavelet feature parameters in the graph are distributed in four different color regions, so these spikes are divided into four categories accordingly, and the average spike in each category is shown in Figure 4d. Define the red high-amplitude spike evoked by neuron 1, the blue sinusoidal spike evoked by neuron 2, the green cosine spike evoked by neuron 3 and the cyan low-amplitude spike evoked by neuron 4. Because the amplitude of the spiking waveform of neuron 1 is higher, the distribution area of the wavelet characteristic parameters of neuron 1 is far away from the other three categories.

For the fourth type of spike, because the amplitude is the lowest, and the base potential is too high, it is considered not to be the neuronal responding activity, but the result of ambient noise. Therefore, the fourth type of spike is not considered in the later discussion. According to the classification algorithm, the number of spiking events of each neuron evoked by twirling manipulation and lifting–thrusting manipulation is shown in Table 2.

In the acupuncture experiment, we use mechanical stimulus with low frequency. In Figure 4a,b, according to the raw data of the two acupuncture manipulations recorded by electrodes, we find that the spiking trains evoked by each acupuncture stimulus are densely clustered. To explore the main factors that evoke the neuronal spiking activity under the conditions of two acupuncture manipulations, we propose the hypothesis that the spike history of neurons is a more important factor in evoking the spiking activity of acupuncture neurons compared to acupuncture stimulus. To verify this hypothesis, we model neuronal spiking activity under two acupuncture manipulation conditions with the coupling GLM. MLE is then used to estimate the coupling parameters of stimulus for each neuron, the self-coupling parameters for its own spike history and the cross-coupling parameters of other neurons’ spike history. The estimated results of the set of coupling parameters for each neuron under two acupuncture manipulations are shown in Table 3.

According to the data in Table 3, Table 4 and Table 5, it can be concluded that under the conditions of the two acupuncture manipulations, the coupling parameters of stimulus of the three neurons are significantly less than the self-coupling parameters and cross-coupling parameters of the spike history. This suggests that stimulus is the inducing factor to evoke neuronal spiking activity, and compared to stimulus, the set of spike history has a larger effect on the spiking activity of each neuron, which is consistent with our previously proposed hypothesis. Therefore, in subsequent analyses of the responding mechanism of different acupuncture manipulations, we mainly consider the self-coupling of the neuron’s own spike history and the cross-coupling of other neurons’ spike history.

Under the conditions of two acupuncture manipulations, when comparing the self-coupling parameters ($h_{11}$, $h_{22}$, $h_{33}$) of three neurons, we find that the self-coupling parameters decrease gradually from neuron 1 to neuron 3. As shown in Figure 4d, neuron 1 exhibits a high-amplitude spike, while neuron 2 and neuron 3 exhibit spikes of lower amplitudes. It is concluded that the self-coupling parameters of neuron’s own spike history decrease as the amplitude diminishes. In addition, for each neuron, the self-coupling parameters of the neuron’s own spike history are smaller than the cross-coupling parameters of other neurons’ spike history. This indicates that the cross-coupling of other neurons’ spike history is the main factor affecting the neuronal spiking activity.

Then, by comparing the two cross-coupling parameters of each neuron, we find that under the condition of twirling manipulation, for neuron 1 with high-amplitude spiking waveform, the cross-coupling parameter $h_{13}$ is significantly larger than $h_{12}$. This indicates that the cross-coupling of spike history of neuron 3 to neuron 1 has a larger effect on the spiking activity of neuron 1. For neurons 2 and 3, the cross-coupling parameters $h_{21}$ and $h_{31}$ are significantly greater than the cross-coupling parameters $h_{23}$ and $h_{32}$, indicating that the cross-coupling of spike history of neuron 1 with a high-amplitude spiking waveform to neurons 2 and 3 has a larger effect on the spiking activity of neurons 2 and 3. The same experimental phenomenon is observed for lifting–thrusting manipulation, but the difference between the two cross-coupling parameters of each neuron is not obvious.

It can be seen that external stimulus is the inducing factor in evoking neuronal spiking activity under acupuncture conditions. Once spikes occur, the coupling relationship of the spike history becomes the main factor of neuronal spiking activity. In addition, the self-coupling parameter decreases as the neuronal spiking amplitude diminishes, and compared with the self-coupling of the neuron’s own spike history, the cross-coupling of other neurons’ spike history is the most important factor in evoking neuronal spiking activity. In the cross-coupling activity of other neurons’ spike history, the higher the amplitude of the neuronal spiking waveform, the greater its cross-coupling parameters.

## 4. Discussion

In this paper, the coupling GLM that we use is the single-neuron model. We add the effect of other neurons’ spike history into the single-neuron model, rather than modeling from the perspective of neural clusters. In subsequent studies, we can try to model the spiking rates of neural ensembles using the black-box probability model, which is a way of describing the spiking activity of neural ensembles from a global perspective. In addition, only the second-order coupling relationship is covered in this model, and the third-order coupling relationship among neurons is not considered, which may have some limitations. Introducing a higher-order coupling relationship in the spiking rate model of neural ensembles may more comprehensively describe the dynamic behavior of neural ensembles.

## 5. Conclusions

In this paper, the affecting factors of neuronal spiking activity are studied, and the coupling GLM is used to describe the neuronal spiking activity evoked by two acupuncture manipulations. Then, we use the MLE to estimate the coupling parameters of stimulus, the self-coupling parameters of the neuron’s own spike history and the cross-coupling parameters of other neurons’ spike history. A comparative analysis is conducted on the set of coupling parameters of three neurons under the conditions of twirling manipulation and lifting–thrusting manipulation. The results show that in the spiking activity of acupuncture neurons, acupuncture stimulus acts as the initial inducing factor, with the coupling of neuronal spike history being the dominant factor. Moreover, under the conditions of two acupuncture manipulations, compared to the cross-coupling activity of other neurons’ spike history, the self-coupling of the neuron’s own spike history has a lesser effect on neuronal spiking activity. Among the cross-coupling activity of other neurons’ spike history, the cross-coupling of neurons with a higher amplitude of spiking waveform plays a more significant role in evoking neuronal spiking activity. The study of acupuncture–response relationships from the perspective of spike history has established a probabilistic model for neuronal spiking events, revealing the responding mechanism of acupuncture. This provides the scientific basis for the selection of acupuncture manipulation and frequency in clinical treatment in the future.

## Figures and Tables

**Table 1:** The estimation of coupling parameters for simulated data.

Coupling	Neuron 1				Neuron 2				Neuron 3			
	$k_{1}$	$h_{11}$	$h_{12}$	$h_{13}$	$k_{2}$	$h_{22}$	$h_{21}$	$h_{23}$	$k_{3}$	$h_{33}$	$h_{31}$	$h_{32}$
True	0.85	1	1.4	0.8	0.9	1.25	1	0.7	0.7	1	0.9	1.2
Estimate	0.843	0.978	1.414	0.787	0.897	1.203	0.980	0.701	0.699	1.002	0.876	1.173

**Table 2:** Number of spiking events of each neuron evoked by two manipulations.

Manipulation	Neuron 1	Neuron 2	Neuron 3
Twirling	879	1032	978
Lifting-Thrusting	1045	849	814

**Table 3:** The estimation of the set of coupling parameters for neuron 1.

Manipulation	$k_{1}$	$h_{11}$	$h_{12}$	$h_{13}$
Twirling	−0.0003	0.2021	0.0970	**0.6380**
Lifting-Thrusting	0.0100	0.1662	0.4911	**0.6562**

**Table 4:** The estimation of the set of coupling parameters for neuron 2.

Manipulation	$k_{2}$	$h_{22}$	$h_{21}$	$h_{23}$
Twirling	0.0027	0.1397	**2.6788**	0.9413
Lifting-Thrusting	−0.0295	−0.0352	**1.5872**	1.5511

**Table 5:** The estimation of the set of coupling parameters for neuron 3.

Manipulation	$k_{3}$	$h_{33}$	$h_{31}$	$h_{32}$
Twirling	0.0003	0.0493	**3.5635**	0.2568
Lifting-Thrusting	−0.0153	−0.0622	**1.6837**	1.2613

## Data Availability

The datasets presented in this article are not readily available because the data are part of an ongoing study.
